# Stunting, food security, markets and food policy in Rwanda

**DOI:** 10.1186/s12889-019-7208-0

**Published:** 2019-07-04

**Authors:** Dave D. Weatherspoon, Steve Miller, Jean Chrysostome Ngabitsinze, Lorraine J. Weatherspoon, James F. Oehmke

**Affiliations:** 1 Agricultural, Food & Resource Economics Department, Michigan State University, Rm. 213C Morrill Hall of Agriculture, 446 W. Circle Dr, East Lansing, MI 48824 USA; 2 Agricultural, Food & Resource Economics Department, Rm. 81 Morrill Hall of Agriculture, 446 W. Circle Dr, East Lansing, MI 48824 USA; 3 Department of Rural Development and Agricultural Economics, College of Agriculture, Animal Sciences and Veterinary Medicine, University of Rwanda, P.O. Box 210, Musanze, 3971 Rwanda; 4 Department of Food Science and Human Nutrition, Michigan State University, 469 Wilson Road, Rm 140 Trout Food Science Bldg, East Lansing, MI 48824-1224 USA; 5 United States Agency for International Development, Bureau For Food Security, Ronald Reagan Bldg., 1300 Pennsylvania Ave. NW, Washington, DC 20229 USA

**Keywords:** Stunting, Rwanda, Policy, Dietary diversity

## Abstract

**Background:**

Over the past two decades, Rwanda has experienced impressive economic growth, resulting in considerable improvements in living standards and poverty reduction. Despite these gains, progress on reducing the level of stunting in smallholder rural children, particularly boys, continues to be a serious concern.

**Methods:**

Policies, dietary diversity and socio-economic factors that may influence stunting in rural Rwandan children were evaluated using a logit model with clustered variance-covariance estimators based on village membership of the household.

**Results:**

Stunting of rural children was found to be multidimensionally related to the child’s gender, weight and age; the dietary diversity, marriage status and education level of the head of household; mother’s height; presence of a family garden or if they owned livestock; environmental factors such as altitude and soil fertility and location relative to a main road en route to a market; and a policy that promoted food production.

**Conclusions:**

Findings suggest that agricultural policies may be subsidizing poor dietary behavior in that the aggregation of production encourages households to sell high quality nutritious food such as fruit and vegetables, for more voluminous amounts of nutritionally substandard goods, hence low dietary diversity. However, it is less clear if rural food markets are capable of supplying diverse and nutritious foods at affordable prices on a consistent basis, resulting in a lack of diversity and hence, low nutrient quality diets. Rwanda’s next round of food security policies should focus on nutrition insecurity with special emphasis on the lack of protein, micronutrients and calories. Multipronged policies and programs focused on income growth, food security, enhanced access to markets and gender-related nutrition risks from inception through 2 years of age in the rural areas are required to improve rural household health outcomes, stunting in particular.

## Background

Despite improvements in reducing world hunger, at least 795 million people globally are afflicted with stunting, including about 159 million children under 5 years of age [1]. Stunting is a nutrition problem, where compromised stature occurs in response to chronic undernutrition – often in conjunction with pre- and post-natal infections [2]. Stunting spurs a vicious cycle of poverty, as stunted mothers are less able to biologically provide sufficient nourishment for the fetus, leading to another stunted generation [3]. The impacts of stunting include reduced cognitive capabilities, language and sensory-motor capacities, mental development, school performance and intellectual capacity. Some research shows that these impacts are difficult to reverse after the age of two [4]. When accompanied by excessive weight gain later in childhood, stunting can lead to obesity and an increased risk of nutrition-related chronic diseases [2, 5, 6]. The long-term economic impacts of a high prevalence of stunting include low adult wages, lost productivity and lower gross domestic product growth, i.e. intransigent poverty [7]. Hoddinott et al. [8] estimated that for every dollar invested in reducing stunting through nutritional programs, $13 - $15 in economic returns is generated for Uganda, Tanzania and Kenya.

Rwanda has one of the highest rates of prevalence of child stunting in the world. The World Health Organization estimates Rwanda’s prevalence of stunting in children under 5 years of age was 37.4% for 2014 and 2015 [9]. This represents a modest decrease from the 2010–2011 prevalence of 44.3%, which trails the reduction of the prevalence of poverty [10]. Smith and Haddad [11] found that stunting is only reduced slightly when there is positive economic growth. Masters et al. [12] suggest that urban food price controls favor urban consumers at the expense of rural poor farmers, hence, little change in malnutrition and stunting is observed. It is estimated that the effects of hunger and undernutrition costs Rwanda approximately US $820 million (504 billion Rwanda francs) annually [13].

The Rwandan government, along with a number of donors, have enacted policy and implemented programs to reduce poverty and malnutrition with the intention of reducing the prevalence of stunting and wasting. Despite significant cross-country evidence linking higher incomes and improvements in stunting and wasting [12], this linkage appears weak at the household level in a number of countries including Mali [14], Burkina Faso [15] and Rwanda [11, 16]). While Rwanda exhibits a negative correlation between wealth and stunting [17], this correlation also varies by geography or perhaps crop selection within Rwanda [16].

While the amount of food consumed is relevant, food intake patterns and dietary diversity are also deemed to be an important contributing factor to malnutrition [18, 19] and stunting, and believed to be contributing factors to the high prevalence of stunting in Rwanda. Stunted children receive nutritionally inadequate and/or unsafe complementary foods. At the same time, less than a fourth of infants 6–23 months of age meet the criteria of dietary diversity and feeding frequency appropriate for their age [20]. Rwanda’s prevalence of stunting differs across sex, geography and wealth. Joint malnutrition estimates from the World Bank, UNICEF and the World Health Organization show that the prevalence of stunting for Rwandan males is 43% compared to 33.4% for females. The prevalence of child (less than 23 months) stunting in rural areas is almost twice that in urban areas, 40.9 and 24.1%, respectively [21]. Design of effective anti-stunting policy thus requires a clear delineation of the various risk and protective factors, and quantification of their effects in the Rwandan context(s).

This paper has two objectives. The first is to determine food intake patterns, dietary diversity, socio-economic risk and protective factors and quantify their influence on stunting in rural Rwandan children. The second is to use this information to inform policy and program design that addresses/ameliorates stunting in rural Rwanda.

### Stunting

Stunting is linear growth retardation accumulated before and after birth. The incidence of stunting in children is generally attributed to maternal undernutrition during pregnancy and child undernutrition within the first 2 years of life [4]. Prevalence is highest in low-resource economies with 75% of stunted children under 5 years of age being located in Sub-Saharan Africa or South Asia [1, 22, 23]. Sub-Saharan Africa has experienced varying levels of stunting with the highest incidence in Eastern Africa [24], where prevalence can be as high as 50% in certain sub-regions within a country [25].

Stunting reflects the child’s food and environmental conditions and is an indicator of the child’s long-term growth and intellectual potential. The UNICEF framework for nutrition intervention is to focus on environmental factors around poverty [24] that impact food availability, consumption and dietary quality since nutrition is integral to child growth and health. The United Nations World Food Programme [26] references food security as a cornerstone of their Millennium Development Goals. However, not all the nutrition sensitive associations with stunting are well understood [23]. As discussed below, research has uncovered a number of correlates that appear to describe individual, environmental and contextual factors associated with stunting.

There is a debate in the literature on whether the effects of stunting are reversable. Adair [4] found that early stunting is reversable, but the majority of children stunted at an early age that persists to age two, will remain stunted throughout their childhood. In her study, using the Longitudinal Health and Nutrition Survey of children from Cebu, Philippines, Adair reports that 63% of children were stunted at age two, and that 42.5% remained stunted at age 12. These findings are supported by later research in Amazonian Bolivia. Godoy et al. [27], using panel data of forager-farmers estimated that the rate of catch-up for stunted children (mean 96 cm) was 0.11 standard deviations per year for children between two and 7 years of age. They also found demographic characteristics, including improved household income and the addition of siblings, increased the rate of catch-up.

#### Individual characteristics

Individual factors that appear to contribute to child stunting include gender, underweight, infections, poor quality of breastmilk from undernourished mothers, and lack of dietary diversity. Wamani et al. [28] found that male children were more stunted than female children in ten Sub-Saharan countries but failed to find any explanatory factors for the differences. Teshome et al. [29] found similar results in Ethiopia but did not conjecture a cause. Mukabutera et al. [30] also found that Rwandan male children are more likely to be underweight and/or stunted. Although not explored in this study, infections (intestinal worms and giardia), sickness (anemia), and the lack of zinc and other nutrients in breast milk have also been associated with stunting [30–33].

Onyango et al. [34] and Divya et al. [35] found support for the measurement of dietary diversity as a strong predictor of the nutritional status of children. M’Kaibi et al. [36] showed that low dietary diversity and associated environmental factors were strongly correlated with stunting in rural Kenya. Adu-Afarwuah et al. [22] and Rah et al. [23] found similar associations in semi-urban Ghana and Bangladesh, respectively. In Rwanda, Niyibituronsa [37] showed a direct link between household dietary diversity and undernutrition, especially referring to a low intake of animal protein sources.

#### Household characteristics

Household level characteristics have been shown to be related to the probability of stunting in children. Education level; mother’s demographics and nutrition and health status; as well as household size, income and assets have all been shown to play a role in determining nutrition and health outcomes for children, in particular, stunting. Higher education and/or nutrition education levels of the head of household and/or the mother have been shown to reduce the probability of stunting in children [38, 39].

The mother’s age, mid-upper arm circumference (MUAC), height, and marital status have all been associated with the stunting in children. Much focus has been given to maternal undernutrition during pregnancy and the early years after birth of the child [4]. The mother’s health status is a good indicator of the nutrition environment the child is reared in, her MUAC provides current nutrition status information and height reflects historical information on whether she was stunted as a child. For example, Ozaltine et al. [40] found an inverse relationship between a mother’s height and the risk of her child being stunted. The marital status of the mother is also commonly included in statistical analysis of stunting with mixed results [41].

Moss et al. [10] show that the prevalence of stunting in Rwanda has not reduced at the same rate as poverty. UNICEF and WHO data show that there is a negative relationship between wealth and stunting worldwide. Habyarimana et al. [41] found that a Rwandan child born to a rich family had a 35.4% lower odds of being stunted compared to a child from a poor household. Godoy et al. [27] found that higher household incomes increased the rate in which the stunted child was able to catch up. Lee et al. [42, 43] interestingly reported a positive correlation with child stunting and overweight in Guatemalan mothers and attributed this association to economic affluence of the surveyed households. Food security is still not assured, even though the household socioeconomic status is not at the lower-end of the spectrum. It is imperative to understand why being overweight is not necessarily associated with nutrient adequacy. Intermingled with these studies is the role the number of children in the household and the overall household size. Assets, particular for rural households, have not been included in most of the studies. Including food related assets, such as gardens and livestock, warrants consideration in the Rwandan context due to their pro gardening and livestock policies (62% and 74% of rural households had gardens and livestock respectively in the current study).

#### Community and environmental characteristics

Geophysical conditions have been shown to impact the prevalence of childhood stunting. Distance to the main road and market have both been used as indicators for how well connected a household is to markets that may be used to purchase and sell food items [44]. Household altitude has been shown to increase the risk of stunting in children [45, 46]. It is unclear if this is a biological relationship or reflecting the lack of available nutritious foods either from own-production or markets. For this reason, alternative indicators are also explored to determine if they are associated with stunting (i.e. soil fertility).

#### Policy

Frongillo et al. [47] findings suggest that macro level policy such as human capital formation, particularly for women, national affluence, and regional expenditures on healthcare influence prevalence of stunting. Milman et al. [48] support these finding and also show that rates of urbanization – which may be associated with affluence, access to health services, access to nutritious foods, etc. – are negatively associated with a higher prevalence of stunting. Investing in policy that addresses stunting has been shown to be just as cost-effective as other public investments [8]. However, given the multidimensionality of stunting, it is difficult to measure policy impacts. More research is needed on the role of economic growth and agricultural development policy impacts on reducing stunting in poor agriculturally based economies [25].

There are two policies that are specific to Rwanda that were developed to address rural poverty and stunting indirectly. Land consolidation and the Integrated Development Program (IDP) policies were implemented to increase agricultural production and efficient land use along with stimulating job creation. Specifically, land consolidation has been credited with increased total production of maize by about five fold; wheat and cassava by about 3 fold; Irish potato, soybean and beans by about 2-fold; and rice by 30% when compared to the base levels in 2007 [49]. Del Prete et al. [50] found that land consolidation had a positive impact on the consumption of roots and tubers, but a reduction in the consumption of meat, fish and fruits. The reduction in dietary diversity due to this policy has major implications for the reduction of stunting for children of rural Rwandan farmers.

The IDP was aimed at reinforcing the capacities of the poor farmers for using land “rationally”, creating jobs and capital through the specialization, land consolidation and harvest processing (storage, transformation factories, etc.). The program also assisted with animal husbandry and infrastructure building [51] and was implemented in about 22% of the villages. The program contributed to land consolidation; soil and water management; crop intensification and livestock development; and the promotion of off-farm activities.

More generally, there may be a policy issue with respect to the prioritization of nutrition in the agricultural sector. Typically, an agricultural sector strategy is developed in terms of targeting the sector growth rate through increased land productivity. Rwanda’s strategies are aligned with this approach [52]. Nutritional concerns then must also fit into this strategy. The land consolidation and IDP policies exemplify the productivity-first approach. Rwanda’s National Agricultural Policy has only two paragraphs directly addressing rural nutrition [52]. If the agricultural policy approach were to ask first “what is it that rural mothers need so that they and their children can lead healthy, productive lives”, then nutritional outcomes might play a more prominent role. For example, there is an easier coordination and alignment between job creation for women (a Rwanda priority) and access to diverse and nutritious food through affordable markets than there is between job creation and home gardening (a Rwanda priority) that typically taxes women and their time allocation [53]. This begs the question of whether Rwanda’s next round of agricultural policies should elevate attention and be more closely aligned with the general objective of the National Food and Nutrition Policy [54]: “to improve the household food security and nutritional status” with emphasis on children under two (p. 25). One indication of the future in Rwanda’s latest sustainable development goals is the newly created National Early Childhood Development Coordination Program, which seeks to reduce the prevalence of stunting to 19% by 2024 through allocating $184 million in programming that addresses nutrition, social protection and agriculture [55]. However, the role that food preferences, poverty and markets play in reducing stunting may still warrant consideration to enhance impact.

## Methods

### Data

The data sample included 3342 records of children under 5 years of age. The sample was derived from a household survey over 714 villages across 30 districts in Rwanda [51]. Data from separate survey instruments were collected for the household, mothers and the children, where child records were linked to mother’s records and mothers’ records were linked to households using unique identification codes.

Only records from rural households were retained, eliminating 533 urban and semi-urban households from the sample. Detecting stunting in ages less than 4 months can be difficult, hence, only those children between the ages of three to 24 months were included in the initial sample. This generated a sample of 870 children. Only 820 of these included a response for the prevalence of stunting. An additional 50 observations were dropped because of incomplete explanatory variables, where absence of the mother’s height accounted for 35 of the dropped observations. A test of equality of the prevalence of stunting of children in the sample and that of those dropped from the sample because of omitted explanatory variables determined the omitted observations does not introduce bias to the sample. The final sample totaled 770 children who ranged from just under 5 months of age to just under 25 months.

### Stunting by gender and age

The estimated prevalence of stunting in this dataset for rural children younger than 2 years of age is approximately 38%. Cross-tabulations reveal that male children are more prone to stunting (Table 1). The odds of a male child between the ages of five and 24 months being stunted is 62.2% higher than that of their female counterparts. This distinction is statistically significant, as indicated with Pearson’s Chi-square test of independence. This finding aligns with Wamani et al. [28] and Teshome et al. [29] who found that boys are more often stunted than girls in Sub-Saharan Africa.

**Table 1:** Prevalence of Stunting by Gender

Stunting	Child Gender		Total
	Female	Male	
No	67.9	56.8	62.3
Yes	32.1	43.2	37.6

*Pearson Chi*^*2*^*(1) Statistic = 10.14*: *Prob. = 0.001*

**Fig. 1:**
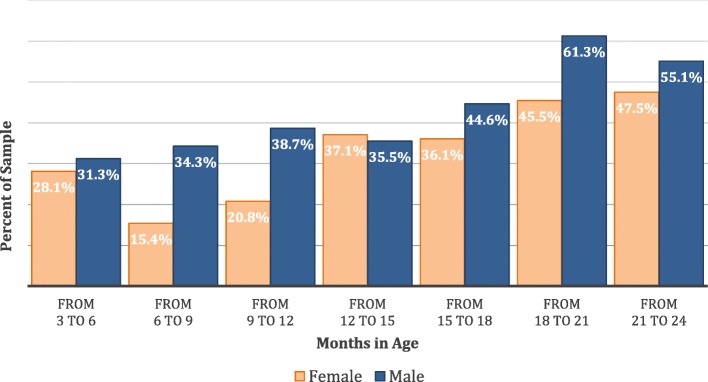
Prevalence of Stunting by Age and Gender

Figure 1 shows that male stunting existed in all age groups but was most prevalent among those between 18 and 24 months in age. However, the proportionate difference between male and female stunting is most pronounced in the 6 to 12 month age groups. Figure 1 also shows that age is associated with an increasing probability of a rural child in Rwanda being stunted. The increasing prevalence appears to counter the expectations that early onset of stunting can be reversed within the first 2 years [27]. This may reflect the mother’s failure to recognize and treat the child’s stunting at an early age to reverse the effects as suggested by Godoy et al. [27], as well as poor complementary feeding. This may also reflect short-term environmental change conditions reflecting consistent food shortages during the child’s first 2 years. Thus, understanding the disparity in the prevalence of stunting between males and females early in age versus later is important for future research.

### Food expenditures and dietary diversity

**Fig. 2:**
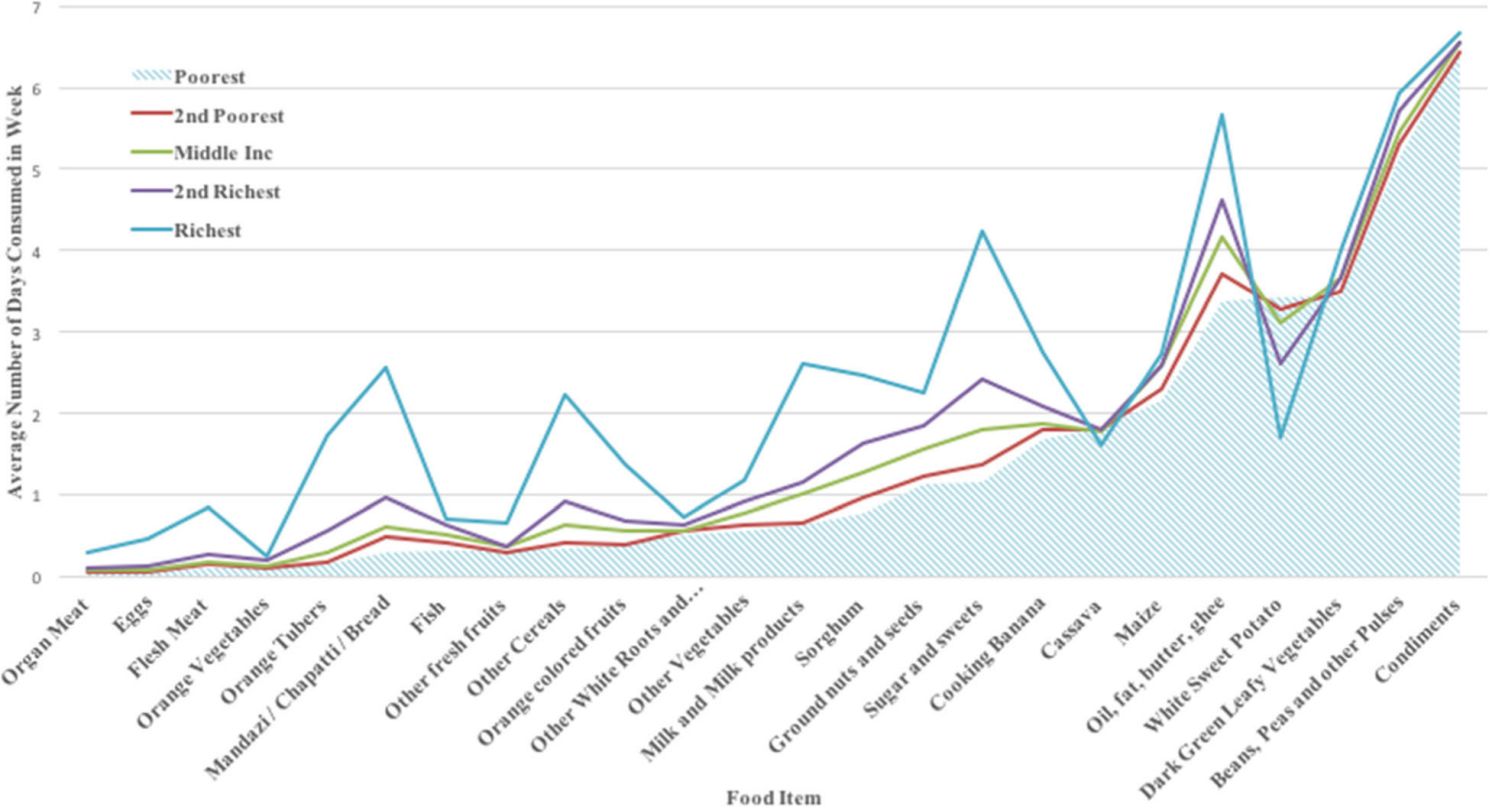
Average Number of Days/Week a Food Item was Consumed by Household Income Group

Rwanda has one of the highest income inequality rates in the world, with a Gini coefficient of 50.4 in 2013 [56]. How income inequalities translate into food expenditures is critical to understanding the persistent prevalence of stunting. Table 2 shows that the richest rural Rwandans spend 2.8, 2.2, 1.9, and 1.46 times more on food than the poorest, 2nd poorest, middle and 2nd richest respectively. The food expenditure share shows how much of all expenditures that food represents for each income group. The poorest spend the most on food relative to all other expenditures followed by the 2nd poorest, 2nd richest^1^ and the middle income groups. Figure 2 reflects how many days per week each food item is consumed by income group. The shaded area shows that the poorest income group shares a similar diet with the next three income groups; they just consume those same food items less often on average. The diet is most diverse for those categorized as being in the highest income group.

**Table 2:** Expenditures and Food Shares by Income Quintiles in Rwandan Francs

	Poorest	2nd Poorest	Middle	2nd Richest	Richest
Total Monthly Expenditure	19,292	29,594	35,707	42,623	102,536
Monthly Food Expenditure	7451	9544	11,178	14,355	20,936
Food Expenditure Share	0.39	0.32	0.31	0.34	0.20

Source: Authors, WFP 2012 DataRF700 = $1 U.S.

Assessing how often various food items are consumed leads to some conclusions. The diet of poor rural Rwandans in this sample was low intake in animal based proteins as well as fruit and vegetables; and high in carbohydrates. Key nutrients that may be limited in such a diet are protein, vitamins A and C, folic acid, calcium and iron. Chronic deficiency is associated with increased risk for diseases such kwashiorkor and marasmus, xerophthalmia, scurvy, neural tube defects, rickets and microcytic anemia respectively [57–59]. Specific concerns for mothers and children in this situation include stunted growth, wasting and nutrient specific symptoms.

### Model

Equation (1) is a logit model with the dependent variable as the probability of stunting.

1 $$ \mathit{\Pr}\left({y}_i\ne 0|{x}_i\right)=\frac{\mathit{\exp}\left({x}_i\beta \right)}{1+\mathit{\exp}\left({x}_i\beta \right)}. $$

The variables x_i_ are the independent variables and represent the various potential risk and mitigating factors described above. In practice, the probability of stunting is not observed and so the left side is replaced with a binary variable that takes the value of one if the child meets the WHO criteria as stunted and zero otherwise. Equation (1) is estimated using maximum likelihood estimation and coefficients are reported as both Logit expressions and as marginal effects, where marginal effects are calculated as p_i_(1-p_i_)B_j_. The clustered variance-covariance estimators are based on village membership of the household as neighborhood effects are likely to impact the independence of observations [60].^2^

### Explanatory variables

Table 3 provides the descriptive statistics for all the explanatory variables included in the analysis. The first two columns provide summary statistics over the entire sample, including sample means and standard deviations. The next two columns break out the sample into those reporting as stunted or not stunted and is provided for comparison. For indicator variables, like male (1 = M, 0 = F), the mean denotes the share of responses meeting that category. The majority of the variable names are self-explanatory but the few that need to be defined are discussed below.

**Table 3:** Summary Statistics and T-Tests

Variable	Sample		Not Stunted		Stunted		T-Test
	Mean	S.D.	Mean	S.D.	Mean	S.D.	Pr(|T| < |t|) = 0
Child Variables							
Stunted (1 = Y,0 = N)	0.38	*(0.48)*	na		na		na
Male (1 = M,0 = F)	0.50	*(0.50)*	0.45	*(0.50)*	0.57	*(0.50)*	0.00
Age in months	14.74	*(5.14)*	14.05	*(5.09)*	15.88	*(5.03)*	0.00
Ill in past 2wks (1 = Y,0 = N)	0.52	*(0.50)*	0.51	*(0.50)*	0.53	*(0.50)*	0.51
Weight (kilograms)	9.17	*(1.64)*	9.51	*(1.67)*	8.62	*(1.44)*	0.00
Household Variables							
Acceptable FCS (1 = Y,0 = N)	0.78	*(0.42)*	0.81	*(0.39)*	0.72	*(0.45)*	0.00
Primary ed. (1 = Y,0 = N)	0.58	*(0.49)*	0.60	*(0.49)*	0.55	*(0.50)*	0.20
Secondary ed. + (1 = Y,0 = N)	0.09	*(0.29)*	0.11	*(0.31)*	0.06	*(0.24)*	0.02
Mother’s age in years	29.13	*(7.43)*	29.10	*(7.39)*	29.18	*(7.50)*	0.88
Mother’s MUAC (in MMs)	259.9	*(26.00)*	261.5	*(27.29)*	257.2	*(23.50)*	0.02
Mother’s height (M)	155.0	*(13.36)*	156.3	*(12.46)*	152.8	*(14.50)*	0.00
Size of household	5.46	*(2.19)*	5.47	*(2.15)*	5.44	*(2.26)*	0.86
Marriage status HH (1 = Y,0 = N)	0.82	*(0.39)*	0.83	*(0.37)*	0.80	*(0.40)*	0.24
Have garden (1 = Y,0 = N)	0.62	*(0.48)*	0.62	*(0.49)*	0.63	*(0.48)*	0.85
Have livestock (1 = Y,0 = N)	0.74	*(0.44)*	0.74	*(0.44)*	0.74	*(0.44)*	0.77
HH per-person food expend’s	2.89	*(3.2)*	2.98	*(3.4)*	2.75	*(2.8)*	0.32
HH per-person non-food expend’s	57.77	*(162.0)*	68.15	*(197.1)*	40.60	*(70.6)*	0.01
Environmental Variables							
Altitude (meters)	1.75	*(0.3)*	1.73	*(0.3)*	1.79	*(0.3)*	0.01
Distance to main road (meters)	4.22	*(3.9)*	4.30	*(4.1)*	4.08	*(3.6)*	0.43
Distance to market (meters)	0.08	*(0.06)*	0.08	*(0.06)*	0.1	*(0.06)*	0.55
Road to market accessible (1 = Y,0 = N)	0.74	*(0.44)*	0.75	*(0.43)*	0.72	*(0.45)*	0.35
Soil fertility index (0?-100?)	0.45	*(0.07)*	0.45	*(0.07)*	0.46	*(0.07)*	0.10
Policy Variables							
Land Consolidation (1 = Y,0 = N)	0.62	*(0.48)*	0.63	*(0.48)*	0.61	*(0.49)*	0.67
IDP Model Village (1 = Y,0 = N)	0.20	*(0.40)*	0.19	*(0.39)*	0.22	*(0.41)*	0.32
N	770		480		290		na

The mid-upper arm circumference (mother’s MUAC), measured in millimeters, is included for the mother, as well as age and height [40]. Marital status is depicted as “married” if the respondent indicated they were married or partnered with a spouse. The alternative is never married, widowed or divorced. Have garden or livestock denotes the presence of a vegetable garden or livestock in the household. Policies for promoting own agricultural production is a component of Rwanda’s strategy for combating malnutrition. Three variables measure access to markets. The variable “road to market accessible” indicates year-round accessibility to the market via road, while “distance to market” measures the distance in meters. Distance to main road is also a measure of mobility to markets and other services provision.

The variable Acceptable FCS is an indicator variable denoting a food consumption score (FCS) of 35 or higher, where FCS measures range from zero to 112. FCS measures reflect the composite quality of the head of household’s dietary intake along multiple dimensions including diversity, frequency and nutritional content [28]. Typical thresholds are:

- 0–21 = Poor, 61% were stunted,
- 21.5–35 = Borderline, 46% were stunted,
- > 35 = Acceptable, 35% were stunted.

FCS scores are calculated based on self-reported consumption over 7 days. Head of household FCS score indicates the immediate food environment the child is located within.

## Results

Prior to running regressions, independent variables were tested against the binary relationship with stunting using simple t-tests on means. T-test were run for each explanatory variable with respect to a child being stunted or not and are shown in the final column of Table 3. The two-tailed test asserts the probability that the stunted and non-stunted means are equal. Tests of equality apply Satterthwaite’s approximation [61] to control for differences in sample variances between the stunted and not stunted samples. The sample size of the summary statistics is 770 (480 not stunted and 290 stunted) and includes all observations used in the subsequent regression estimations.

The child’s individual characteristics reveal that gender, age and weight of child subjects were all significantly different between rural stunted and non-stunted children in Rwanda. The head of household characteristics show that their food consumption score (i.e. dietary diversity) and their level of education significantly influence stunting in children. In addition, the household’s per capita non-food expenditures and the mother’s height and MUAC were significantly different for those households that had stunted children. The environmental factors that are significantly different are the household’s altitude and the soil fertility level around the household.

### Logit model results

Table 4 shows logit model results of bivariate relationships to stunting. A clustered sandwich estimator was applied based on 424 villages represented in the regression sample. STATA 13.1 was used for the analysis. The cluster estimator relaxes the assumption that every child sampled is independent, but rather allows that individuals within a village may share attributes that impact the prevalence of stunting within that village. Other specifications, including Huber/White/sandwich and grouped logit estimators were also estimated but not reported. The model estimates of these alternative specifications were found to be consistent with the Logit model with clustered standard errors shown.

**Table 4:** Logit Estimations Results (Dependent variable is Stunting: Clustered Std. Errors)

Stunted	All Subjects (between 3 and 24 months)			Male Subjects			Female Subjects		
	Coef.	dydx		Coef.	dydx		Coef.	dydx	
Child Variables									
Male (1 = M,0 = F)	1.314	0.197	***						
Age in months	0.364	0.055	***	0.384	0.058	***	0.371	0.052	***
Ill in past 2wks (1 = Y,0 = N)	−0.153	−0.023		−0.257	− 0.039		− 0.099	− 0.014	
Weight (kilograms)	−1.329	− 0.199	***	−1.435	− 0.218	***	− 1.259	− 0.176	***
Household Variables									
Acceptable FCS (1 = Y,0 = N)	−0.586	−0.088	***	−0.465	− 0.071		−0.831	− 0.116	***
Primary ed. (1 = Y,0 = N)	−0.322	−0.048		− 0.205	−0.031		− 0.459	−0.064	
Secondary ed. + (1 = Y,0 = N)	− 0.822	−0.123	**	−0.938	− 0.142		−0.923	− 0.129	*
Mother’s age in years	0.005	0.001		−0.012	−0.002		0.017	0.002	
Mother’s MUAC (in MMs)	0.0035	0.0004		0.005	0.001		0.0	0.0	
Mother’s height (M)	−0.019	−0.003		−0.017	− 0.003	*	− 0.016	−0.002	
Size of household	0.041	0.006		0.054	0.008		0.047	0.007	
Marriage status HH (1 = Y,0 = N)	−0.35	−0.053		0.073	0.011		−0.749	−0.105	**
Have garden (1 = Y,0 = N)	0.43	0.065	**	0.344	0.052		0.536	0.075	*
Have livestock (1 = Y,0 = N)	0.034	0.005		−0.434	−0.066		0.634	0.089	*
HH per-person food expend’s^a^	0.021	0.003		−0.047	−0.007		0.115	0.016	
HH per-person non-food expend’s^a^	−0.001	−0.0		0.0	0.0		−0.0036	−0.001	
Environmental Variables									
Altitude (1000 m)	0.499	0.075	*	0.455	0.069		0.559	0.078	
Distance to main road (1000 m)	−0.024	− 0.004		−0.053	− 0.008		0.0	0.0	
Distance to market (1000 m)	−2.280	−0.342		− 2.898	− 0.439		−1.696	−0.237	
Road to market accessible (1 = Y,0 = N)	−0.408	−0.061	*	−0.574	− 0.087	*	− 0.016	−0.002	
Soil fertility index (0?-100?)	2.351	0.353	*	3.641	0.553	*	0.647	0.09	
Policy Variables									
Land Consolidation (1 = Y,0 = N)	−0.265	−0.039		−0.414	− 0.063		−0.176	− 0.025	
IDP Model Village (1 = Y,0 = N)	0.407	0.061	*	0.323	0.049		0.492	0.069	
Constant	6.673	na	**	8.282	na	***	5.895	na	
Number of obs	770			384			386		
Wald Chi2	180.00		***	93.98		***	84.26		***
Pseudo R2	0.310			0.325			0.318		

(Std. Err. adjusted for 364 clusters in v_code)

“*”, “**” and “***” denotes coefficient significance at the 10, 5 and 1% levels, respectively

^a^indicates coefficients in 1000s

Table 4 shows the Logit regression and impact factor estimate results for three identically specified models. The first model includes 770 rural children, while the second and third models specify relationships underlying the 384 male and 386 female children, respectively. As indicated, the models explain a significant amount of the variation in the probability a rural child in Rwanda will be stunted, with numerous significant coefficients. Psuedo R-squares ranged from .31 to .325 and the Wald Chi-squared statistic of model fit indicated significance at the one-percent level. Link tests of each model [62], suggests the models, based on these independent variables, are properly specified in the current Logit form. Other tests of Pearson correlation coefficients also indicated no presence of multicollinearity in the independent variables. The coefficient columns take the standard Logit model interpretation where non-linear associations cloud the extent of association between the independent variables and the probability of stunting. The marginal effects coefficients (dy/dx) are scale-comparable as marginal effects on the probability of being stunted.

#### Child characteristics

Interpreting the findings and consistent with other findings for this age group, male children were more likely to be stunted than their female counterparts [28–30]. Based on marginal effects coefficients (dy/dx), males were nearly 20% (19.7%) more likely to suffer stunting. The findings also suggest that age is positively and significantly associated with stunting. Consistent with Fig. 1, for every additional month in age, the probability of a child being stunted increases by about 5.5%. Child weight is also an important consideration. World Health Organization [2] states that the mortality risk of children who are even mildly underweight is increased, and severely underweight children are at even greater risk of stunting. We found that the higher the weight of the child, the lower the probability of child stunting, in that every kilogram of weight reduced the chance of stunting by 20%. However, Rwanda is not a food poor environment and has not been subject to food shocks in recent years, though food diversity and security is a concern [63]. Therefore, we hypothesized that food intake patterns and diversity, relative to stunting, may be a more significant factor [23, 64] than food shocks, as indicated by the significantly strong negative association with acceptable FCS scores and stunting.

### Household characteristics

Several household and head of household characteristics were tested, but few proved statistically significant as a predictor of childhood stunting. The education attainment of the head of household is negatively associated with the child’s stunting level. About 58% of the respondents indicated having some or completed primary education. Just under 33% indicated no education, while about 10% indicated some or more vocational or secondary education. The base case is no schooling. Children in households where the head of household has more than some exposure to secondary education were about 12% less likely to be stunted. Male children did not appear to share the benefit of the head of household’s education level, in terms of stunting, as female children, however. This finding was difficult to comprehend, as joint tests of significance of the two education variables showed significance for the whole sample and the female sample, but not the male sample.

Except for mother’s height, which was significant and negatively associated with male child stunting, mother’s age, MUAC and total household size were not significantly related to child stunting. The male child stunting association with mother’s height was however weak with the coefficient remaining negative across all three models. About 82% of respondents indicated being married or with a partner. While not significant overall, it appears that marital status has asymmetric effects on stunting outcomes between male and female children, where having both parents in the household appears to be associated with reduced stunting outcomes in female children.

Rwanda’s National Food and Nutrition Policy strategic initiatives aim to improve household access to and promote subsistence agriculture, including household vegetable gardening and livestock rearing [54]. Accordingly, about 62.3% of households indicated having a vegetable garden. A vegetable garden may augment already sufficient household diets or may be a defense to food privation. Hence, the positive coefficient on the presence of a vegetable garden may signal a means of subsistence under privation. However, this association is relatively weak at a 10% level of significance. The presence of small livestock also has a positive coefficient for all subjects but is not significant. This association is weakly significant for female children. However, concerning male children, the sign is reversed, though not significant. One may interpret the negative association as an indicator of household behaviors toward own production of food. Households are thought to sell garden and livestock production to subsidize purchased foods, where purchased foods favor low-cost staples. That is, a nutritionally diverse diet is being substituted with a less diverse diet that is low in animal protein and high in starch.

The final household questions address expenditures, where household expenditure categories were aggregated into food and non-food expenditures. Expenditures were transformed into per-person expenditures by dividing by the size of the household. Neither food nor non-food expenditures entered the models significantly. Interestingly food expenditures enter the overall children and female children models with a positive coefficient, non-food expenditures enter with a negative coefficient and vice versa for male children. Because food and non-food expenditures make up total expenditures, the combination may proxy for total household income. A joint test of significance of the two expenditure categories indicates that total per-person expenditures was insignificant to stunting outcomes in all samples except for the female sample.^3^

### Community and environmental characteristics

Locational factors were also tested. Accessible roads to a market was significant and negatively related to stunting for the overall and male child models. Male children are 8% less likely to be stunted if the household has good access to a road leading to the market. Living at higher altitudes increased the chances of the child exhibiting stunting but was not significant over the gender sub-samples. Alternatively, households in villages with higher levels of soil fertility tended to disproportionately impact male children. In this regard, higher soil fertility was associated with a higher prevalence of stunting, though the association may also be related to other locational attributes.

### Policy

Rwanda’s IDP policy was implemented from 2010 to 2013 in support of Rwanda’s National Human Settlement Policy. The policy aim at the household level is to assist poor farmers in allocating their household resources more efficiently and at the community level to assist with creating jobs in rural communities. The policy directly reinforced the land consolidation policy by focusing on soil and water management; crop intensification and livestock development; and the promotion of off-farm income generating activities. This policy was significant and positive for the overall model only. Households that were located in districts where this policy was implemented were 6% more likely to have stunted children than those that were not in IDP areas. At the implementation stage of the policy, IDP area farmers experienced land shortage and/or they found it difficult to produce on both the old and new land due to the distance between them. The additional resources required to produce on both properties may have negatively affected the diets of those who were included in the IDP policy area.

## Discussion and conclusions

This study characterizes rural Rwandan household, food and health, community and policy environments and analyzes factors that are associated with stunting in children from just under 5 months to 2 years of age. Stunting can begin as a result of intrauterine growth retardation associated with poor nutrition and continue through the critical period of 24 months post- delivery and beyond. Ultimately, generations are negatively impacted due to a vicious cycle that ensues [3, 6, 7]. Therefore, our findings are fundamental to understanding whether economic growth and agricultural development policy have the potential to affect stunting [11, 12]. The current analysis confirms that although economic growth is occurring at an impressive rate, stunting remains a major problem in rural areas of Rwanda. As found in other studies, Rwandan boys are more likely to be stunted than girls [28–30].

Policymakers cite reducing the prevalence of childhood stunting as a primary motivator for creating policies and programs that increase household income and/or reforming land policy [8, 47, 48, 50]. Evidence from the current study indicates that the land reform policy had no significant impact on the prevalence of stunting in the rural areas. The IDP Model Village policy was associated with a small but significant likelihood of higher stunting in the rural areas. In fact, these policies may be subsidizing poor dietary behavior in that the aggregation of production is now encouraged and output markets exist that did not previously. This allows the household to sell high quality nutritious food such as fruit and vegetables, for more voluminous amounts of low-nutritionally-substandard goods. However, it is less clear if rural food markets are capable of supplying diverse and nutritious foods at affordable prices on a consistent basis, resulting in a lack of diversity and hence, low nutrient quality diet. Results show that the economic threshold to improve nutrition is extremely high; the poor households would have to consume a diverse diet equivalent to the second highest income group, essentially doubling their current food expenditures. This study also questions the efficacy of some of the existing Rwandan National Food and Nutrition Policies. Specifically, the one cow and food security garden plot policies appear to be effective in households engaging and adopting the policy. However, the adoption of this policy has not translated into improved diets, but increased household sales of agricultural products, particularly animal-based proteins. Of greater concern is the fact that the presence of a garden or livestock increased the risk of stunting in those households. This suggests that there is a divergence between program intent (improved nutrition in the context of agricultural growth) and program outcome (improved income through sales of nutritious foods).

Rwanda’s next round of agricultural growth policies must actually be more nutrition specific; focusing on the lack of protein, micronutrients and calories. The IDP and Land Consolidation policies encourage the selling of high quality nutritious food products, but they do not provide a mechanism that enables the household to retain or purchase an equally nutritious bundle of food items from the market. Our findings question whether households use the gardens or livestock policies for financial gain, not nutrition gain, as evident in the increased risk of stunting with gardens and livestock. Therefore, one important strategy might be to use nutrition education to enhance/support the personal use of some of the food produced or raised instead of only focusing on financial gain. Further research needs to delineate if and how personal production relates to personal use/consumption. Market accessibility is a protective factor presumably from better access to food markets, but the role of rural output markets in child stunting needs to be better understood and policies must factor the market effects into any new programs [44]. In particular, improved access to output markets and crop consolidation to achieve economies of scale in the absence of improved rural food markets is expected to lead to higher production of cash crops, lower production of food for home consumption, and inadequate opportunities to purchase nutritious foods in the market. Hence, infrastructure and policy support for rural food market development could be the critical missing components in Rwanda’s nutrition strategy. Importantly, the rural food markets could accelerate agricultural sector growth by encouraging the demand for higher-value fruits and vegetables, thereby generating “win-win” outcomes with positive impacts on both growth and nutrition.

Rwanda’s latest sustainable development goals seek to reduce the prevalence of stunting to 19% by 2024 through the newly created National Early Childhood Development Coordination Program. The $184 million program addresses nutrition, social protection and agriculture, but may still fail at addressing the complicated role that food preferences, poverty and markets play in reducing stunting. Dietary diversity is an important consideration that needs to be integrated into any intervention strategy [18, 19]. Therefore, a promising approach is for the agricultural sector to elevate dietary diversity through an agriculture sector objective. This could be accomplished through complementary government and private sector investments that increase food production, improve rural food markets and promote rural nutrition education to enhance consistent year-round consumption of diverse foods in rural households.

## Data Availability

The data that support the findings of this study are available online at the National Institute of Statistics of Rwanda at http://www.statistics.gov.rw/datasource/comprehensive-food-security-and-vulnerability-and-nutrition-analysis-survey-cfsva

## Abbreviations

FCS: Food consumption score
IDP: Integrated Development Program
MUAC: Mid-upper arm circumference
UNICEF: United Nations International Children’s Emergency Fund
USAID: United States Agency for International Development
WHO: World Health Organization

## Footnotes

1 The slight increase in food expenditure share for the 2nd richest may reflect an upgrade in dietary quality and the addition of more processed foods.

2 Alternative model specifications, including Probit and standard sandwich-type robust standard errors. However, the Logit model with clustered variance generally exhibited better overall fit, but the statistical significance of slope coefficients were largely unchanged across model specifications.

3 Other specifications were tried including share of total expenditures allocated to food and total expenditures. The coefficient on share of expenditures on food tended toward the positive, which may be expected if we interpret that lower income households will tend to spend a higher share of expenditures on food than more affluent households do.
